# Differentiating Hodgkin Lymphoma and Sarcoid Reaction in Subsequent FDG-PET/CT: A Case Report and Literature Review

**DOI:** 10.1055/s-0043-1777694

**Published:** 2023-12-26

**Authors:** Obayda Rabei, Ula Al-Rasheed, Mohammed Alrammahi, Akram Al-Ibraheem

**Affiliations:** 1 Department of Nuclear Medicine, King Hussein Cancer Center (KHCC), 202 Queen Rania St., P.O. Box 1269, Amman, Jordan

**Keywords:** FDG-PET/CT, Hodgkin lymphoma, sarcoid reaction, sarcoidosis, diagnostic challenge

## Abstract

Sarcoidosis is frequently associated with various hematological and solid tumors; it can be discovered by chance during tumor evaluations. Sarcoidosis can occur before some cancers, coexist with others, or be diagnosed 1 to 2 years later. Sarcoid reaction affecting hilar and mediastinal lymph nodes can pose a diagnostic challenge in patients with histopathological confirmation of Hodgkin lymphoma who are being evaluated using fluorodeoxyglucose-positron emission tomography computed tomography (FDG-PET/CT) scan because it cannot be easily distinguished from lymphoma infiltration. The presence of an increase or persistence of a prominent activity on a follow-up FDG-PET/CT scan after chemotherapy treatment for lymphoma that is associated with a complete metabolic response in the site of the primarily diagnosed lymphomatous disease is highly suggestive of concurrent sarcoidosis and necessitates careful assessment to avoid unnecessary therapy.

## Introduction

Sarcoidosis is multisystem granulomatous disease of unknown etiology with pulmonary and extrapulmonary manifestations. It is most commonly diagnosed in the third and fourth decades of life worldwide, and it is frequently reported in Sweden, Denmark, and people of African origin. Brincker described the association of sarcoidosis with lymphoma for the first time, discovering a significantly higher than expected association between sarcoidosis and lymphoma of all types, with an approximately 11.5-fold increased risk. 1

The sarcoidosis-lymphoma syndrome refers to lymphoma that develops 1 to 2 years after a sarcoidosis diagnosis. 1 As it was discovered that sarcoidosis patients can also develop hematologic malignancies and solid tumors, such as lung and liver cancer, the name of the syndrome has been changed to sarcoidosis-malignancy syndrome. 2 Paraneoplastic sarcoidosis refers to sarcoidosis that develops concurrently with or within a year of the diagnosis of hematologic malignancy or solid tumor. 2 The third entity is sarcoid reaction, which occurs when noncaseating granulomata of the regional lymph nodes or the organ of tumor involvement are present without the systemic manifestations of sarcoidosis. 2

Granulomatous reactions are commonly linked to lymphoma. There have been reports of sarcoid-like reactions occurring in up to 13.8% of patients with Hodgkin lymphomas (HL) and 7.3% of patients with non-Hodgkin lymphomas (NHL). 3 4 5 Granulomatous reactions are typically observed in the same lymph node or organ as the lymphoma, but they can also occur in other hematopoietic organs. Sarcoid-like reactions may be the predominant histological finding with few tumor cells, particularly in HL or T-cell lymphomas, making lymphoproliferative disorder difficult to diagnose. 6

Fluorodeoxyglucose-positron emission tomography computed tomography ([ ^18^ F] FDG-PET/CT) is the primary imaging modality for staging HL and monitoring treatment response. 7 Moreover, interim PET/CT demonstrated significant prognostic value and is regarded as a potent predictor of HL patient outcome 8 ; however, its sensitivity is hindered by active inflammation and granulation tissues, which are known to accumulate FDG. Active sarcoidosis has a high FDG uptake and, as a result, can be a diagnostic pitfall for malignancies such as lymphomas.

In this case report, we present a difficult-to-confirm case of synchronous HL and sarcoid-like reaction detected during staging imaging. The HL demonstrated a complete metabolic response to four cycles of the ABVD chemotherapy protocol (doxorubicin, bleomycin, vinblastine, and dacarbazine), whereas the sarcoid reaction demonstrated a clinically and radiologically significant aggravated reaction.

## Case Presentation

A 17-year-old male patient with nonsignificant past medical history presented complaining of 2 months history of progressive painless left neck swelling, there was no other concerning symptoms, no history of fever, weight loss, or shortness of breath.

**Fig. 1:**
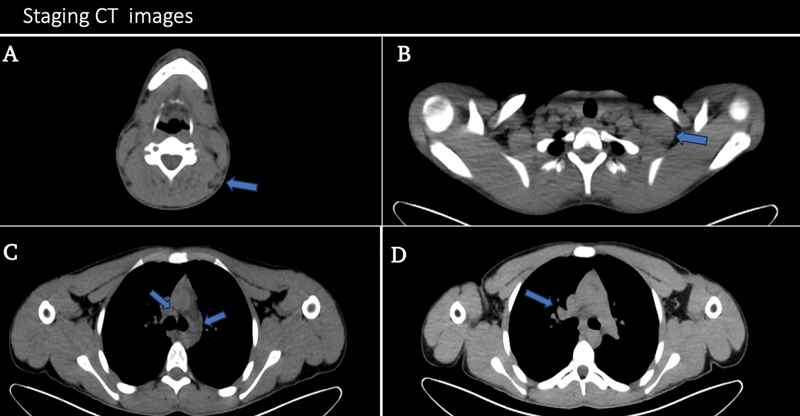
A 17-year-old male patient diagnosed with Hodgkin lymphoma. ( **A** ) Cross-sectional computed tomography (CT) slice showing an abnormally enlarged left cervical lymph node level Va ( *blue arrow* ). ( **B** ) Cross-sectional CT slice showing an abnormally enlarged left supraclavicular matted lymph nodes ( *blue arrow* ). ( **C** , **D** ) Cross-sectional CT slices showing bilateral hilar and mediastinal lymph nodes ( *blue arrows* ).

**Fig. 2:**
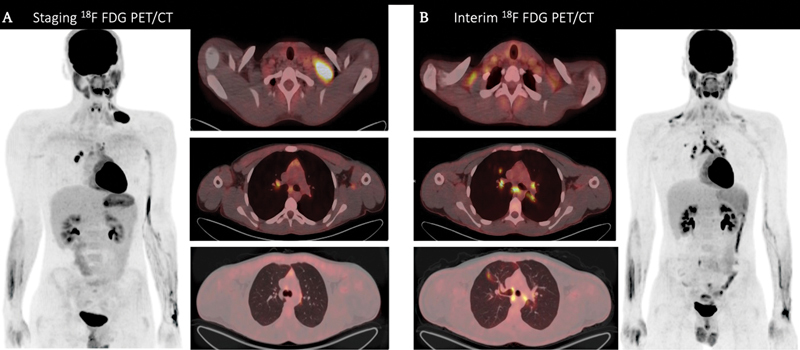
A 17-year-old male patient diagnosed with Hodgkin lymphoma. ( **A** ) Imaging from staging fluorodeoxyglucose-positron emission tomography computed tomography ( ^18^ F-FDG-PET/CT) scan reveals hypermetabolic left cervical, left supraclavicular, and bilateral mediastinal lymph nodes (more so on the right side) with normal lung parenchyma bilaterally ( *left image* : anterior maximum intensity projection image from ^18^ F-FDG-PET; *right images* : axial fused PET/CT images). ( **B** ) Images from interim ^18^ F-FDG PET/CT scan demonstrating complete metabolic resolution of the hypermetabolic left cervical and left supraclavicular lymph nodes, with persistence of bilateral mediastinal and hilar lymph nodes with interval increase in number and note of diffuse bilateral supraclavicular brown fat FDG uptake along with interval development of mildly hypermetabolic right upper lobe pulmonary ground glass opacity ( *right image* : anterior maximum intensity projection image from ^18^ F-FDG-PET; left images: axial fused PET/CT images).

A CT scan was performed, which revealed enlargement of the lymph nodes in the left neck, including the left cervical, left supraclavicular, and bilateral mediastinal lymph nodes ( Fig. 1 ). A biopsy of the left supraclavicular lymph nodes was performed, and histopathology revealed that the tumor cells were positive for MUM-1, CD30, CD15, PAX-5, and CD20 (occasional cells positive). They were CD79a and CD3 negative. A staging PET/CT scan was performed, which revealed multiple hypermetabolic aggregated/matted left cervical lymphadenopathies at levels II, III, and IV (supraclavicular region), with the largest one measuring about 4.8 × 2.3 cm and maximum standardized uptake value (SUVmax-15.2), as well as hypermetabolic few bilateral mediastinal and hilar small lymph nodes ( Fig. 2 ).

After four cycles of ABVD, an interim PET/CT scan was performed to assess the patient's response to therapy. The PET/CT scan revealed complete metabolic resolution of the left cervical and left supraclavicular lymph nodes; however, the few bilateral symmetrical mediastinal and hilar lymph nodes were still noted with interval increases in their number, metabolic activity, and slight size increment compared to the staging scan, as well as interval development of mildly enlarged lymph nodes ( Fig. 2 ).

This pattern of persistent symmetrical hypermetabolic mediastinal and hilar lymphadenopathy in the context of complete metabolic resolution of previous biopsy-proven cervical and supraclavicular lymphomatous lymphadenopathy, with interval right upper lung manifestations, raised the possibility of a pre-exiting active granulomatous disease manifested by hypermetabolic bilateral mediastinal and hilar lymphadenopathy exacerbated by chemotherapy. Nonetheless, histopathological confirmation remains the gold standard for final diagnosis.

## Discussion

HL is a rare lymphatic neoplasm, but it is one of the most common cancers in young adults. 9 A low number of malignant cells derived from B-lymphocytes and an extensive inflammatory microenvironment characterizes the disease.

Thanks to new therapy regimens and strategies, the prognosis of HL has steadily improved over the last several decades. Originally considered an almost incurable disease in the 1940s, HL has evolved into a type of cancer with one of the best prognoses. 10

Currently, the main therapeutic approaches are based on two chemotherapy regimens: ABVD and BEACOPP (doxorubicin, bleomycin, vinblastine, and dacarbazine) (bleomycin, etoposide, doxorubicin, cyclophosphamide, vincristine, procarbazine, and prednisone). The latter improves survival progression but increases long-term toxicity, which affects fertility and increases secondary leukemia. 11 12 Currently, [ ^18^ F] FDG-PET/CT is the main imaging modality for staging and monitoring response to treatment in HL.

The relationship between malignancy and sarcoidosis occurs in three different presentations, the first concurrence is in patients with hematologic malignancies. It includes the sarcoidosis-lymphoma syndrome, which refers to the development of lymphoma at least 1 to 2 years after the diagnosis of sarcoidosis. It also includes patients with sarcoidosis who develop other hematologic malignancies.

In addition, patients with cancer and hematologic malignancies who develop sarcoidosis form a second subset of concurrence. Patients with solid tumors who develop sarcoidosis and oncologic patients who develop sarcoidosis; the neoplasms most commonly associated with sarcoidosis in this type include melanoma and nonmelanoma skin cancer, cervix, liver, lung, testicles, and uterus. 2

The third type of malignancy-related sarcoidosis occurs when sarcoidosis manifests as a paraneoplastic syndrome for the associated cancer, specifically when the cancer is discovered concurrently with or within 1 year of the sarcoidosis diagnosis, or vice versa.

Antineoplastic treatment of either hematologic or solid tumors has also been observed to either cause the onset or flare-up of sarcoidosis activity. 13 Sarcoid reactions are sometimes associated with cancer. Sarcoid reactions are the development of noncaseating epithelioid cell granulomas in patients who do not meet the systemic sarcoidosis criteria. This sarcoid-like reaction has been most commonly observed in oncologic patients in the lymph nodes draining the cancer. It is especially common in testicular cancer 14 and lymphoma. 5 Sarcoid reactions occur in 4.4% of carcinomas, 13.8% of Hodgkin disease patients, and 7.3% of NHLs. 14

Sarcoid reactions are caused by antigenic factors derived from tumor cells, which trigger an immunological hypersensitivity response that results in the formation of epithelioid-cell granulomas. 5 Sarcoid reactions may be a marker of an immunologically mediated antitumor response of activated macrophages by T-lymphocytes, and there is evidence that patients with sarcoid reactions have a better prognosis in Hodgkin disease. 5

In a small prospective study, Papanikolaou and Sharma conclude that sarcoidosis and lymphoma, primarily NHL, can coexist, with sarcoidosis usually coming before lymphoma. Because many of the symptoms of sarcoidosis and lymphoma are the same, histological confirmation of malignancy is required. Sarcoidosis, while uncommon, can complicate the course of lymphoma. Respiratory symptoms, high angiotensin-converting enzyme levels, bilateral hilar lymphadenopathy in asymptomatic subjects, and lung disease were all prominent features of sarcoidosis. Despite the limitations of this study, the above-described features should alert clinicians to pursue a second diagnosis, in order either to properly diagnose malignancy in the sarcoidosis setting or to save cancer patient from a potentially toxic treatment escalation due to a presumed lymphoma recurrence. 15

Accurate interpretation of FDG-PET/CT findings is critical for determining HL prognosis and selecting appropriate treatment. FDG, on the other hand, frequently accumulates in benign processes such as infection or granulomatous inflammation, resulting in false positive results. Relatively symmetrical FDG-avid bilateral hilar and mediastinal lymph nodes in a “lambda”, “Christmas Tree,” or “butterfly” distribution pattern, which we defined as “sarcoid-like” distribution pattern, are not uncommonly observed on FDG-PET/CT but with different interpretation among different physicians, especially for the lymph nodes demonstrating high SUV (SUV > 2.5). 16

In this case report, we want to highlight the importance of taking into consideration the possibility of presence of active sarcoidosis in a pattern of FDG avid symmetrical bilateral mediastinal and hilar small lymph nodes when assessing patients with HL to avoid false-positive FDG-PET/CT interpretation.

FDG-PET/CT post-chemotherapy interim scan evaluation demonstrating the status of persistent FDG avid symmetrical bilateral mediastinal and hilar lymphadenopathy with interval progressive metabolic and morphologic features as well as interval lung parenchymal changes, associated with complete resolution at the site of the biopsy proven lymphomatous infiltration in the left lower cervical area seen on staging PET/CT, support and strength the suggestion of co-existing sarcoidosis in the mediastinal and hilar lymphadenopathy that was aggravated metabolically and morphologically by the chemotherapy. A study of five patients with histopathologically confirmed sarcoid-like reactions that developed after cancer remission; they found that time interval between the diagnosis of malignancy and sarcoid like reactions was 9 to 78 months. 17 There was insufficient evidence in the literature on the timeline follow-up FDG-PET/CT to differentiate both entities. In equivocal cases, we recommend a minimum of 3 to 6 months for follow-up PET/CT to distinguish both entities. However, histological confirmation is still the gold standard approach for differentiating sarcoidosis from true lymphomatous disease infiltration.

## Conclusion

In conclusion, the presence of granulomatous sarcoidosis in HL patients can pose a diagnostic challenge on staging FDG-PET/CT scans. Interim FDG-PET/CT scans have the potential to differentiate between the two conditions as they show different patterns of response. Nuclear medicine physicians and clinicians must be aware of this potential coexistence because it can influence treatment decisions and follow-up strategies for patients.
